# Simple, rapid and accurate molecular diagnosis of acute promyelocytic leukemia by loop mediated amplification technology

**DOI:** 10.18632/oncoscience.114

**Published:** 2014-12-27

**Authors:** Orietta Spinelli, Alessandro Rambaldi, Francesca Rigo, Pamela Zanghì, Elena D'Agostini, Giulia Amicarelli, Francesco Colotta, Mariadomenica Divona, Claudia Ciardi, Francesco Lo Coco, Giulia Minnucci

**Affiliations:** ^1^ Hematology and Bone Marrow Transplant Unit, Azienda Ospedaliera Papa Giovanni XXIII, Bergamo, Italy; ^2^ DiaSorin SpA, Gerenzano (VA), Italy; ^3^ Department of Biomedicine and Prevention, University Tor Vergata, Roma, Italy; ^4^ Fondazione Santa Lucia, Rome, Italy

**Keywords:** APL, PML-RARA, molecular diagnosis, LAMP

## Abstract

The diagnostic work-up of acute promyelocytic leukemia (APL) includes the cytogenetic demonstration of the t(15;17) translocation and/or the PML-RARA chimeric transcript by RQ-PCR or RT-PCR. This latter assays provide suitable results in 3-6 hours. We describe here two new, rapid and specific assays that detect PML-RARA transcripts, based on the RT-QLAMP (Reverse Transcription-Quenching Loop-mediated Isothermal Amplification) technology in which RNA retrotranscription and cDNA amplification are carried out in a single tube with one enzyme at one temperature, in fluorescence and real time format. A single tube triplex assay detects bcr1 and bcr3 PML-RARA transcripts along with GUS housekeeping gene. A single tube duplex assay detects bcr2 and GUSB. In 73 APL cases, these assays detected in 16 minutes bcr1, bcr2 and bcr3 transcripts. All 81 non-APL samples were negative by RT-QLAMP for chimeric transcripts whereas GUSB was detectable. In 11 APL patients in which RT-PCR yielded equivocal breakpoint type results, RT-QLAMP assays unequivocally and accurately defined the breakpoint type (as confirmed by sequencing). Furthermore, RT-QLAMP could amplify two bcr2 transcripts with particularly extended PML exon 6 deletions not amplified by RQ-PCR. RT-QLAMP reproducible sensitivity is 10^−3^ for bcr1 and bcr3 and 10^−^2 for bcr2 thus making this assay particularly attractive at diagnosis and leaving RQ-PCR for the molecular monitoring of minimal residual disease during the follow up. In conclusion, PML-RARA RT-QLAMP compared to RT-PCR or RQ-PCR is a valid improvement to perform rapid, simple and accurate molecular diagnosis of APL.

## INTRODUCTION

Acute Promyelocytic Leukemia (APL) is a subtype of Acute Myeloid Leukemia (AML) characterized by a specific morphology of the tumor cells [1] and by a balanced reciprocal translocation t(15;17) which fuses the PML gene on chromosome 15 to the *RARA* gene on chromosome 17 [2-6]. The onset of the disease is also frequently characterized by a severe coagulopathy that exposes patients to high risk of fatal bleedings [7]. Interestingly, the incidence of APL and particularly its prevalence amongst AMLs seem to be different among ethnical groups and geographic areas and the disease has been reported as particularly frequent in developing countries of Latin America [8]. Beyond the evaluation of a blood and/or marrow smear, the diagnostic work-up of this leukemia includes conventional or fluorescent in situ hybridization (FISH) cytogenetic [9] and molecular detection of the PML-RARA fusion gene [10]. In addition, the diagnosis of APL may be obtained by immunofluorescence staining using a specific anti-PML antibody that recognizes a distinctive nuclear distribution pattern of the translocated PML protein [11, 12]. The latter does not, however, distinguish the variable PML-RARA isoforms whose precise identification is essential for successive molecular monitoring of minimal residual disease during follow-up [10, 13].

The accuracy and speed of the diagnostic work up is mandatory to start as soon as possible the life-saving treatment with all-trans retinoic acid (ATRA) combined with anthracycline or arsenic trioxide (ATO). These strategies have dramatically changed the natural history of this disease and converted APL from a highly fatal into a highly curable leukemia [14]. Unfortunately, a significant proportion of patients still do not benefit from these highly effective treatments due to an undue delay of diagnosis that may lead to life-threatening coagulopathy or because the onset itself of the disease is characterized by a fatal central nervous system (CNS) hemorrhage [15]. Based on current guidelines of the LeukemiaNet panel [16], immediate recommended actions should be based on the sole morphologic suspicion of APL and these include the start of ATRA and supportive care therapy and sending a blood marrow sample to a reference laboratory for molecular testing. Confirmation of diagnosis at the genetic level is considered essential for patient eligibility to ATRA and/or ATO-based treatments [16].

Identification of PML-RARA chimeric transcripts using Reverse Transcriptase-Polymerase Chain Reaction (RT-PCR) or Real-time Quantitative PCR (RQ-PCR) is performed in specialized laboratories that deliver the diagnosis in approximately 3-6 hours [13, 17, 18]. A simple and rapid molecular assay for APL may improve patient management and, as such, should ideally be feasible even in routine clinical laboratories outside specialized centers or in developing countries where significant improvements in the outcome of APL patients has been already achieved thanks to networking initiatives [19].

The loop-mediated isothermal amplification (LAMP) technology is an innovative non-PCR based nucleic acid amplification method that rapidly amplifies DNA or RNA targets under isothermal conditions [20]. LAMP is performed using strand-displacement polymerase and does not require Taq DNA polymerase or thermal cycling. The potential diagnostic applications of this technology are remarkable and rapidly expanding, particularly in the field of infectious [21] and hematologic diseases [22, 23]. With this background, we show that an improved version of LAMP technology allows a rapid, simple and accurate diagnosis of APL.

## RESULTS

### Development of a new quantitative, fluorescent LAMP assays (RT-QLAMP) for PML-RARA

**Figure 1:**
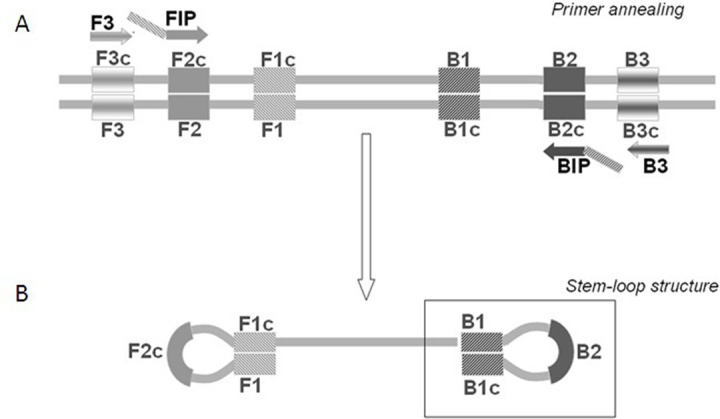
The LAMP technology principle At a constant reaction temperature FIP and BIP primers are extended on the target DNA and the newly synthesized DNA chains are then displaced by extension of F3 and B3 (Panel A). The displaced product generates a “stem-loop structure” which represents the starting structure for a LAMP reaction (Panel B).

**Figure 2:**
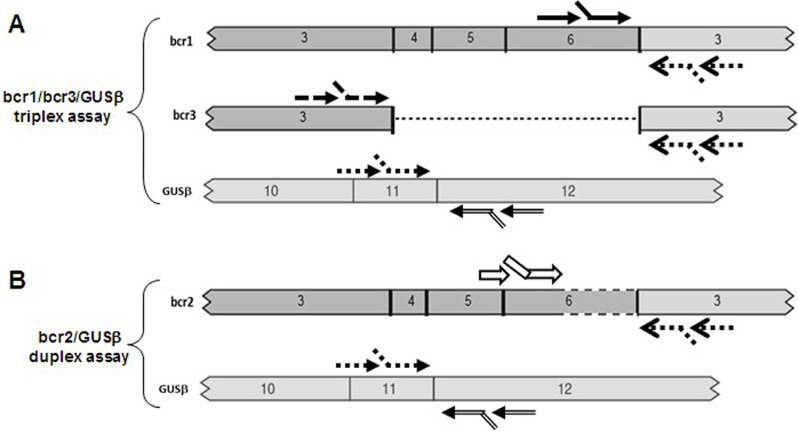
PML-RARA RQ-LAMP assays design Panel A: schematic representation of the triplex assay which can amplify in a single tube bcr1, bcr3 and GUSβ. It provides the molecular diagnosis in 95% of APL cases. Oligonucleotide primers position for amplification are indicated. Panel B: schematic representation of the duplex assay for the amplification of bcr2 isoform, present in 5% of APL cases.

**Figure 3:**
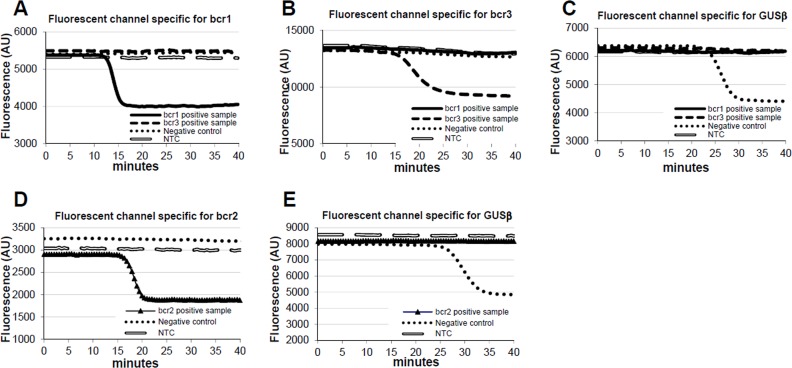
PML-RARA RQ-LAMP amplification plots Representative amplification curves of bcr1 (Panel A), bcr3 (Panel B) and bcr2 (Panel D) PML-RARA positive cases obtained with triplex and duplex assays, respectively. Negative controls are represented in Panel C and E in which the only GUSβ gene is amplified.

The loop-mediated isothermal amplification (LAMP) technology is able to rapidly amplify DNA at constant temperature due to the utilization of a DNA polymerase with an additional strand displacement activity [20] (see Figure 1 and methods section). We modified this method by introducing fluorescent oligonucleotides and a new polymerase with both RNA retrotranscription and DNA amplification activity allowing single tube target amplification with one enzyme at one temperature, in a fluorescence and real time format (RT-QLAMP). We applied this new methodology to the identification of PML-RARA chimeric gene in APL. Two RT-QLAMP assays were designed to detect the 3 PML-RARA chimeric transcripts (bcr1, bcr2 and bcr3). One assay was developed for the simultaneous amplification in a single tube of bcr1 and bcr3, and GUSB housekeeping gene as an internal control (triplex assay) (Figure 2A). This assay may provide molecular diagnosis in 95% of APL cases. To set up this triplex RT-QLAMP assay we used NB4 cell line (bcr1 positive) and APL patients (bcr3 positive) RNA. Representative fluorescence quenching curves for bcr1, bcr3 and GUSB are shown in Figure 3A-B and C respectively. All 30 bcr1 and 30 bcr3 positive RNA samples tested were correctly amplified by the RT-QLAMP triplex assay.

A separate assay was set up for the bcr2 and GUSB amplification (duplex assay) (Figure 2B). Representative fluorescence quenching curves are in Figure 3D-E for bcr2 and GUSB respectively. All 6 APL bcr2 samples (as assessed by RT-PCR) were correctly detected by the RT-QLAMP duplex assay.

**Figure 4:**
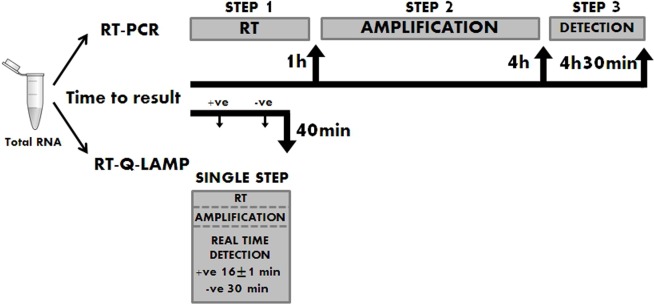
Comparison between RT-PCR and RT-QLAMP workflow RT-QLAMP assays provide results in 16±1 min for PML-RARA positive samples and in 30 minutes for negative samples. Conventional RT-PCR takes about 4.5 hours to have the analysis completed.

The average detection time for all APL patients of PML-RARA transcripts with RT-QLAMP assays was 16±1 minutes while PML-RARA negativity (amplification of GUSB only) could be assessed in non APL samples in 30 minutes (Figure 3A-E). Thus, PML-RARA RT-QLAMP assays provide molecular diagnosis of APL at least 4 hours before RT-PCR (Figure 4).

RT-QLAMP assays were also tested on 699 replicates of RNA from 8 cell lines negative for PML-RARA fusion gene showing a 100% specificity (confidence interval: 99.47-100). All replicates proved negative for PML-RARA and, as expected, positive for the internal control GUSB.

### Validation of PML-RARA RT-QLAMP assays on clinical samples

**Figure 5:**
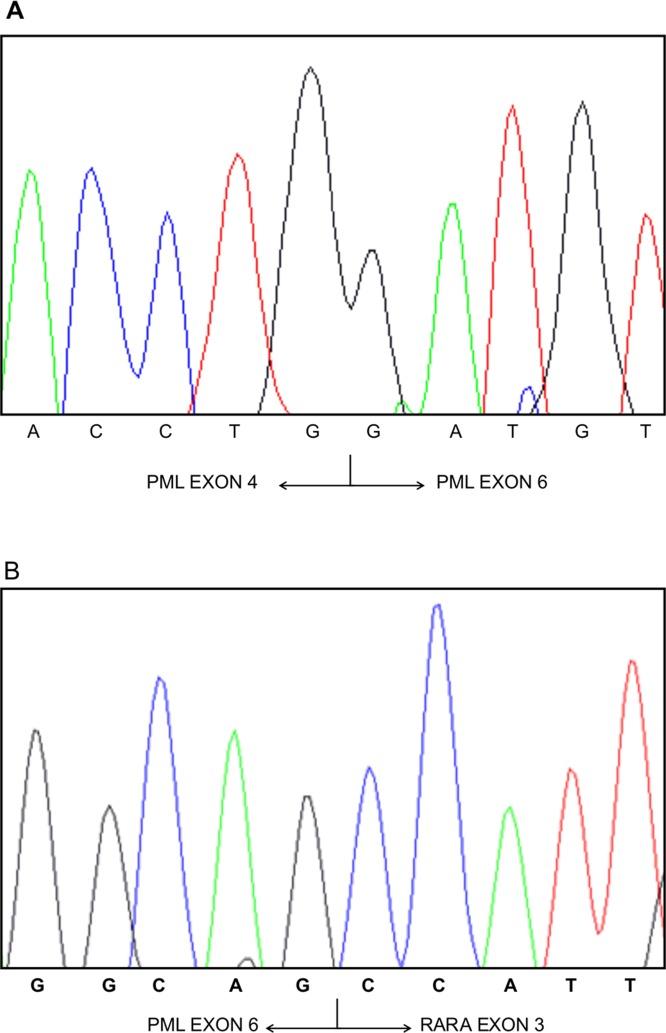
PML-RARA variant with exon 5 deletion Sanger sequencing of the peculiar PML-RARA variant (sample 2514BG126, Table 1) with deletion of PML exon 5 (Panel A) and juxtaposition of PML exon 6 and RARA exon 3 of bcr1 variant (Panel B).

Seventy-three samples collected from two different sets of molecularly confirmed APL patients were selected and tested in blind by RT-QLAMP to confirm the diagnosis and the breakpoint type. Both PML-RARA RT-QLAMP assays correctly identified the 73 APL samples as well as 81 APL-negative controls run in parallel (100% sensitivity and specificity). In 11 cases for whom the RT-PCR assay failed to clearly discriminate between bcr1 or bcr2 (the two chimeric transcripts may be different for only a small number of deleted nucleotides) the breakpoint type could be correctly defined by RT-QLAMP as bcr1 in nine cases and bcr2 in one as confirmed by direct sequencing (Table 1). In addition, in one case (sample 2514BG126, Table 1) RT-PCR incorrectly identified as bcr3 an unusual breakpoint (due to deletion of PML exon 5, Figure 5A-B) which instead was accurately defined as bcr1 by RT-QLAMP and DNA sequencing. Finally, two bcr2 cases which could not be amplified by RQ-PCR [13] because of particularly long deletions of PML exon 6 were properly amplified by RT-QLAMP (data not shown).

**Table 1:** Samples with ambiguous breakpoint definition by conventional RT-PCR tested by RT-QLAMP and confirmed by Sanger sequencing

Sample	RT-PCR result	RT-Q-LAMP result	Sequencing result
5322BG104	Equivocal bcr1/bcr2	bcr1	bcr1
15113BG105	Equivocal bcr1/bcr2	bcr1	bcr1
5734BG120	Equivocal bcr1/bcr2	bcr1	bcr1
2514BG126	bcr3	bcr1	bcr1
BM1547/12	Equivocal bcr1/bcr2	bcr1	bcr1
BM1666/12	Equivocal bcr1/bcr2	bcr1	bcr1
BM1685/12	Equivocal bcr1/bcr2	bcr1	bcr1
BM1722/12	Equivocal bcr1/bcr2	bcr1	bcr1
BM1724/12	Equivocal bcr1/bcr2	bcr1	bcr1
BM560/13	Equivocal bcr1/bcr2	bcr1	bcr1
BM512/12	Equivocal bcr1/bcr2	bcr2	bcr2

### Detection limit of PML-RARA RT-QLAMP assays

**Figure 6:**
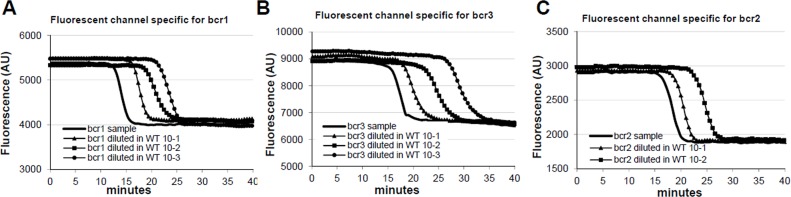
PML-RARA RQ-LAMP limit of detection RT-QLAMP amplification curves of bcr1, bcr3 and bcr2 variants from NB4 cell line RNA (Panel A) or Patient RNA (Panel B and C, respectively) undiluted and serially diluted in HL60 RNA. Bcr1 and bcr3 limit of detection sensitivity is 10^−3^, while bcr2 sensitivity is 10^−2^. Interestingly dilutions of target are detected in a quantitative fashion.

The limits of detection sensitivity of the RT-QLAMP were evaluated on serial positive RNA dilutions (from 10^−1^ to 10^−5^) in wild type HL-60 RNA. Sensitivity of bcr1 was 10^−3^ in 98% of 367 replicates (Figure 6A and Table 2), 10-3 for bcr3 (patient derived RNA, Figure 6B) and 10^−2^ in 100% of replicates for bcr2. Using plasmids with cloned breakpoints, RT-QLAMP assays were able to detect up to 20 DNA copies in 95% and 10 copies in 73% of replicates for bcr3 (Table 2) and 20 DNA copies in 99% of replicates for bcr2 (Figure 6C and Table 2). Interestingly, in all cases, the amplification of transcripts serially diluted in wild type RNA showed a quantitative pattern (Figure 6).

**Table 2:** Performance of triplex and duplex assays in identifying PML-RARA variants when artificially diluted in HL60 RNA

Assay	Variant	Sample type	Dilution Factor inHL60	% detection	Replicates Number
Triplex	Bcr1	NB4 RNA	10^−3^	97,8	367
		NB4 RNA	10^−4^	36,7	30
		Bcr1 patient RNA	10^−3^	100	4
	Bcr3	Bcr3 containing plasmid	20 copies/reaction	95,2	290
		Bcr3 containing plasmid	10 copies/reaction	73	37
		Bcr3 patient RNA	10^−3^	100	4
Duplex	Bcr2	Bcr2 containing plasmid	20 copies/reaction	99,6	229
		Bcr2 patient RNA	10^−2^	100	2

## DISCUSSION

The novel RT-QLAMP assays here proposed to detect the PML-RARA chimeric transcripts allow target amplification in a single tube with one enzyme at one temperature, in a real time-fluorescence format and offers some advantages over conventional RT-PCR and RQ-PCR at diagnosis. Indeed, RT-QLAMP assays do not require a separate retro-transcription of RNA into cDNA before the amplification process since the DNA polymerase displays also reverse transcriptase activity, along with strand displacement activity that allows isothermal amplification. All in all, RT-QLAMP assays amplify chimeric PML-RARA transcripts in approximately 15 minutes starting from RNA, providing molecular diagnosis of APL at least 4 hours before RT-PCR and one hour before RQ-PCR. In addition, unlike conventional RT-PCR, the amplification products obtained by RT-QLAMP do not require any further manipulation since they are visualized as different fluorescent quenching signals specific for each of the three PML-RARA transcripts and for the internal control that allow real time monitoring and isoforms discrimination.

The multiplex nature of RT-QLAMP has the additional distinguished feature of “true” internal control (GUSB) that is co-amplified with PML-RARA transcripts, allowing for accurate control of RNA extraction, RNA quality and amplification conditions, including incorrect assay set up or presence of inhibitors. The closed tube format from RNA to results also prevents DNA spillages and risk for cross-contamination.

Although no firm relationship between fusion variants and outcome has been reported [24] the correct definition of isoforms at diagnosis is essential for successive minimal residual disease monitoring by Real-time Quantitative PCR (RQ-PCR) [16]. For this aspect, RT-QLAMP may offer some significant advantage compared to RT-PCR and RQ-PCR since it accurately defined the transcript type in 11 cases undefined by RT-PCR, 1 case misclassified by RT-PCR and in 2 patients bearing a bcr2 rearrangement not amplified by the widely used EAC (Europe Against Cancer Program) RQ-PCR assay [13].

RT-QLAMP assay could be easily applied even in very small hospitals within non specialized laboratories thus avoiding any diagnostic delay related to the shipment of patients' samples to reference hematological centers.

The LAMP technology was firstly developed as an isothermal reaction in which DNA amplification was detected by naked eye as precipitates formation during the reaction [21]. The RT-QLAMP for PML-RARA isoforms detection in the fluorescent, real time format described here or even the original, turbidity-based LAMP assay [22], could be also easily applied in developing countries to ensure rapid, simple and accurate diagnosis to all APL patients [25].

Although the RT-QLAMP can detect the presence of PML-RARA transcripts down to 10^−3^ (bcr1 and bcr3) or 10^−2^ (bcr2) positive RNA diluted in wild type RNA, this assay cannot be used in its present form as a quantitative assay for the detection of minimal residual disease during the follow-up. Therefore, at this time, the conventional nested PCR reaction or a RQ-PCR assay remain the reference methods for this purpose [26-28]. Nonetheless, the possibility to generate a quantitative Q-LAMP assay for detection of PML-RARA transcripts is under evaluation.

## METHODS

### Patients and samples

In this study we used total RNA derived from 93 clinical samples (73 APL, 10 B cell Chronic Lymphocytic Leukemia (B-CLL), 4 B precursor Acute Lymphoid Leukemia, (ALL), 4 Acute Myeloid Leukemia (AML), 1 Polycythemia Vera (PV), 1 Chronic Myeloid Leukemia (CML), and 61 healthy donors. These clinical samples were collected from 1995 to 2012 at two different clinical sites (Bergamo and Rome) from subjects who gave their informed consent. Total RNA was extracted by phenol-chloroform extraction [29] or RNeasy mini kit (Qiagen, Hilden, Germany) from mononuclear cells isolated by Ficoll-Hypaque gradient centrifugation and lysed in guanidinium iso-thiocyanate (GITC). Samples were tested in blind.

### Cell Lines

The following human cell lines were used: NB4 [t(15;17) APL], HL60 (AML, bearing the BCOR mutation) [30], KASUMI-1 [t(8;21) RUNX1-RUNX1T1 AML], K562 [t(9;22) BCR-ABL1 p210-positive chronic myeloid leukemia in erythroid blast crisis], TOM-1 [t(9;22) BCR-ABL1, p190-positive ALL], 697 [t(1;19) TCF3-PBX1, ALL], RS411 and MV4 [both t(4;11) KMT2A-AFF1, positive ALLs], REH [t(12;21) ETV6-RUNX1, ALL] [10].

### Conventional identification of PML-RARA chimeric transcripts

Identification of PML-RARA chimeric transcript was routinely performed on diagnostic total RNA by Reverse Transcriptase-Polymerase Chain Reaction (RT-PCR). The PML-RARA different transcripts were searched in two separate PCR reactions, one for bcr1 and bcr2 transcript variants and one for bcr3 variant as previously described [10, 18]. Samples positivity for PML-RARA transcripts was assessed by agarose gel electrophoresis of the amplification products. Amplification bands were detected by intercalating dye molecules and UV excitation. The RNA integrity and the efficiency of the retro-transcription step were evaluated for each cDNA by the amplification of the wild type ABL1 gene in a separate PCR reaction. RQ-PCR was performed according to EAC (Europe Against Cancer program) protocol [13].

### RT-QLAMP

The loop-mediated isothermal amplification (LAMP) was initially described by Notomi to amplify DNA under isothermal conditions [20]. The LAMP reaction (Figure 1) is a non-PCR isothermal method for rapid amplification of nucleic acids. It is based on the use of four primers specifically designed to recognize six distinct regions on the target genes: a pair of outer primers (F3 and B3) and a pair of inner primers (FIP and BIP) presenting a tag complementary to a downstream region in the opposite strand of the target (F1 and B1). Each primer is 16-42 bp long. F3 and FIP primers are complementary to the PML region upstream the break point, whereas BIP and B3 primers to the RARA region downstream the break point. The reaction is conducted at a constant temperature in the presence of a DNA polymerase with strand displacement activity. The FIP and BIP primers anneal and are extended on the target DNA and the newly synthesized DNA chains are then displaced by extension of F3 and B3. The displaced product generates a “stem loop structure” which represents the starting structure for a classical LAMP reaction. By addition of labeled probes it is possible to monitor in real time the amplification onto the Liaison IAM instrument (DiaSorin, SpA). At time zero the reaction, excited with appropriate wavelength UV light, emits a maximum fluorescence. As a function of amplification, the fluorescence deceases exponentially thanks to the natural quenching effect of newly synthetized amplicons. The threshold minute is the minute at which the sample fluorescence reaches the 50% of quenching and is correlated with the amount of target present in the reaction. The RT-QLAMP (Reverse Transcriptase-Quantitative LAMP) represents a further improvement of LAMP, allowing amplification of target starting directly from RNA thanks to the additional reverse transcriptase activity embedded in the DNA polymerase enzyme. The PML-RARA RT-QLAMP consists of two fluorescent multiplex assays, one specific for the most frequent transcripts (bcr1 and bcr3) and one for the rarer bcr2 (information available upon request). Retrotranscription and amplification precede both at one constant temperature in a closed-tube format. To control extraction procedure, RNA integrity, reaction functionality and absence of inhibitors, both assays also detect the endogenous GUSβ housekeeping RNA as internal control. The amplification curves become visible in about 15 minutes for PML-RARA positive samples. The implementation of 3 probes specific for the bcr1, bcr2, bcr3 transcripts, labeled with 3 different fluorochromes characterized by distinct wavelengths of emission, allows not only to detect the PML-RARA translocation, but also to distinguish the precise isoform.
